# H275Y Mutant Pandemic (H1N1) 2009 Virus in Immunocompromised Patients

**DOI:** 10.3201/eid1704.101429

**Published:** 2011-04

**Authors:** Christian Renaud, Alexandre A. Boudreault, Jane Kuypers, Kathryn H. Lofy, Lawrence Corey, Michael J. Boeckh, Janet A. Englund

**Affiliations:** Author affiliations: Fred Hutchinson Cancer Research Center, Seattle, Washington, USA (C. Renaud, A.A. Boudreault, L. Corey, M.J. Boeckh, J.A. Englund);; Seattle Children’s Hospital, Seattle (C. Renaud, J.A. Englund);; University of Washington, Seattle (J. Kuypers, L. Corey, M.J. Boeckh, J.A. Englund);; Washington State Department of Health, Shoreline, Washington, USA (K.H. Lofy)

**Keywords:** Influenza, oseltamivir, immunocompromised host, diagnosis, treatment outcome, antiviral drug resistance, oncology, viruses, research

## Abstract

Most oseltamivir-resistant pandemic (H1N1) 2009 viruses have been isolated from immunocompromised patients. To describe the clinical features, treatment, outcomes, and virologic data associated with infection from pandemic (H1N1) 2009 virus with H275Y mutation in immunocompromised patients, we retrospectively identified 49 hematology–oncology patients infected with pandemic (H1N1) 2009 virus. Samples from 33 of those patients were tested for H275Y genotype by allele-specific real-time PCR. Of the 8 patients in whom H275Y mutations was identified, 1 had severe pneumonia; 3 had mild pneumonia with prolonged virus shedding; and 4 had upper respiratory tract infection, of whom 3 had prolonged virus shedding. All patients had received oseltamivir before the H275Y mutation was detected; 1 had received antiviral prophylaxis. Three patients excreted resistant virus for >60 days. Emergence of oseltamivir resistance is frequent in immunocompromised patients infected with pandemic (H1N1) 2009 virus and can be associated with a wide range of clinical disease and viral kinetics.

## MEDSCAPE CME

Medscape, LLC is pleased to provide online continuing medical education (CME) for this journal article, allowing clinicians the opportunity to earn CME credit.

This activity has been planned and implemented in accordance with the Essential Areas and policies of the Accreditation Council for Continuing Medical Education through the joint sponsorship of Medscape, LLC and Emerging Infectious Diseases. Medscape, LLC is accredited by the ACCME to provide continuing medical education for physicians.

Medscape, LLC designates this Journal-based CME activity for a maximum of 1 *AMA PRA Category 1 Credit(s)*^TM^. Physicians should claim only the credit commensurate with the extent of their participation in the activity.

All other clinicians completing this activity will be issued a certificate of participation. To participate in this journal CME activity: (1) review the learning objectives and author disclosures; (2) study the education content; (3) take the post-test and/or complete the evaluation at **www.medscape.org/journal/eid**; (4) view/print certificate.

**Release date: March 23, 2011; Expiration date: March 23, 2012**

## Learning Objectives

Upon completion of this activity, participants will be able to:

- Distinguish clinical characteristics associated with treatment-resistant pandemic (H1N1) 2009
- Analyze outcomes of patients infected with treatment-resistant pandemic (H1N1) 2009
- Identify the rate of H275Y mutation development among patients with pandemic (H1N1) 2009 infection in the current study
- Evaluate the virology of treatment-resistant pandemic (H1N1) 2009

## MDSCAPE CME EDITOR

**Karen L. Foster,** Technical Writer/Editor, *Emerging Infectious Diseases. Disclosure: Karen L. Foster has disclosed no relevant financial relationships.*

## MEDSCAPE CME AUTHOR

**Charles P. Vega, MD,** Associate Professor; Residency Director, Department of Family Medicine, University of California, Irvine. *Disclosure: Charles P. Vega, MD, has disclosed no relevant financial relationships.*

## AUTHORS

*Disclosures:* ***Christian Renaud, MD, MSc; Alexandre A. Boudreault; Jane Kuypers, PhD; Kathryn H. Lofy, MD;*** *and* ***Lawrence Corey, MD,*** *have disclosed no relevant financial relationships.* ***Michael J. Boeckh, MD,*** *has disclosed the following relevant financial relationships: received grants for clinical research from Adamas, GlaxoSmithKline, and Roche; served as an advisor or consultant for Baxter HealthCare Pharmaceuticals and Roche/Genentech, Inc.* ***Janet A. Englund, MD,*** *has disclosed the following relevant financial relationships: received grants for clinical research from Adamas, ADMA, Chimerix, MedImmune Inc., and Novartis Pharmaceuticals Corporation; served as an advisor to US Attorney General, District of Alaska.*

## Earning CME Credit

To obtain credit, you should first read the journal article. After reading the article, you should be able to answer the following, related, multiple-choice questions. To complete the questions and earn continuing medical education (CME) credit, please go to **www.medscape.org/journal/eid**. Credit cannot be obtained for tests completed on paper, although you may use the worksheet below to keep a record of your answers. You must be a registered user on Medscape.org. If you are not registered on Medscape.org, please click on the New Users: Free Registration link on the left hand side of the website to register. Only one answer is correct for each question. Once you successfully answer all post-test questions you will be able to view and/or print your certificate. For questions regarding the content of this activity, contact the accredited provider, CME@medscape.net. For technical assistance, contact CME@webmd.net. American Medical Association’s Physician’s Recognition Award (AMA PRA) credits are accepted in the US as evidence of participation in CME activities. For further information on this award, please refer to http://www.ama-assn.org/ama/pub/category/2922.html. The AMA has determined that physicians not licensed in the US who participate in this CME activity are eligible for *AMA PRA Category 1 Credits*™. Through agreements that the AMA has made with agencies in some countries, AMA PRA credit is acceptable as evidence of participation in CME activities. If you are not licensed in the US and want to obtain an AMA PRA CME credit, please complete the questions online, print the certificate and present it to your national medical association.

## Article Title: H275Y Mutant Pandemic (H1N1) 2009 Virus in Immunocompromised Patients

## MEDSCAPE CME Questions

**1. You are seeing a 50-year-old man being treated for acute myeloid leukemia. He complains of 3 days of fever, chills, fatigue, and wet cough. You suspect influenza, which has been more prevalent in your area over the last month, but you are concerned regarding possible antiviral resistance in this high-risk patient. Which of the following statements regarding the clinical characteristics of patients in the current study who have resistant pandemic (H1N1) 2009 is most accurate?**A. Only adults, not children, had pandemic (H1N1) 2009 with the H275Y mutationB. Most cases of resistant pandemic (H1N1) 2009 were generated from contact with other infected patientsC. Most cases of resistant pandemic (H1N1) 2009 were diagnosed more than 5 days after the onset of symptomsD. Lymphopenia was present in nearly half of patients with the H275Y mutation**2. A rapid polymerase chain reaction (PCR) test is positive for the patient in question number 1. Which of the following statements regarding outcomes of patients with the H275Y mutation in the current study is most accurate?**A. No patients diedB. The H275Y mutation was associated with higher levels of virulenceC. Viral shedding was prolonged, even after clinical recoveryD. No patient receiving only oseltamivir experienced clinical recovery**3. What was the rate of mutation development among all infected patients in the current study?**A. 4%B. 16%C. 28%D. 50%**4. Which of the following statements regarding the virology of cases in the current study is most accurate?**A. Pandemic (H1N1) 2009 was detected in all tissues in the patient with the most severe illnessB. There was generally no discrepancy between genotyping from nasal wash and bronchoalveolar lavage samplesC. The combination of oseltamivir plus rimantadine prevented the development of the H275Y mutationD. The initial viral load had no effect on the emergence of resistance

## Activity Evaluation

**1:** The activity supported the learning objectives.

Strongly Disagree				Strongly Agree
1	2	3	4	5
2. The material was organized clearly for learning to occur.				
Strongly Disagree				Strongly Agree
1	2	3	4	5
3. The content learned from this activity will impact my practice.				
Strongly Disagree				Strongly Agree
1	2	3	4	5
4. The activity was presented objectively and free of commercial bias.				
Strongly Disagree				Strongly Agree
1	2	3	4	5
