# Supplementary material for: H275Y Mutant Pandemic (H1N1) 2009 Virus in Immunocompromised Patients
Source: Emerg Infect Dis. 2011 Apr;17(4):653–60. doi: 10.3201/eid1704.101429 (PMC3290123; doi:10.3201/eid1704.101429)
Supplement: Related texts

# Research article: H275Y Mutant Pandemic (H1N1) 2009 Virus in Immunocompromised Patients

**DOI:** 10.3201/eid1704.101429

**Authors:** Christian Renaud, Alexandre A. Boudreault, Jane Kuypers, Kathryn H. Lofy, Lawrence Corey, Michael J. Boeckh, Janet A. Englund

**Affiliations:** Author affiliations: Fred Hutchinson Cancer Research Center, Seattle, Washington, USA (C. Renaud, A.A. Boudreault, L. Corey, M.J. Boeckh, J.A. Englund);; Seattle Children’s Hospital, Seattle (C. Renaud, J.A. Englund);; University of Washington, Seattle (J. Kuypers, L. Corey, M.J. Boeckh, J.A. Englund);; Washington State Department of Health, Shoreline, Washington, USA (K.H. Lofy)

## Abstract

Most oseltamivir-resistant pandemic (H1N1) 2009 viruses have been isolated from immunocompromised patients. To describe the clinical features, treatment, outcomes, and virologic data associated with infection from pandemic (H1N1) 2009 virus with H275Y mutation in immunocompromised patients, we retrospectively identified 49 hematology–oncology patients infected with pandemic (H1N1) 2009 virus. Samples from 33 of those patients were tested for H275Y genotype by allele-specific real-time PCR. Of the 8 patients in whom H275Y mutations was identified, 1 had severe pneumonia; 3 had mild pneumonia with prolonged virus shedding; and 4 had upper respiratory tract infection, of whom 3 had prolonged virus shedding. All patients had received oseltamivir before the H275Y mutation was detected; 1 had received antiviral prophylaxis. Three patients excreted resistant virus for >60 days. Emergence of oseltamivir resistance is frequent in immunocompromised patients infected with pandemic (H1N1) 2009 virus and can be associated with a wide range of clinical disease and viral kinetics.

The development of antiviral drug resistance in influenza viruses affects patient care. Concerns for worldwide spread of resistant virus are growing (*1*). Approximately 300 patients with oseltamivir-resistant pandemic (H1N1) 2009 virus have been reported to the World Health Organization, with the complexity of treatment and consequences of infection well described (*2**–**5*). Millions of oseltamivir doses have been stockpiled worldwide, representing one of the major interventions to contain and mitigate the impact of influenza and potentially offer treatment to large numbers of patients (*6**,**7*). The efficacy and cost of pharmacologic interventions to contain oseltamivir-resistant virus are of major concern.

Most resistant pandemic (H1N1) 2009 viruses have been detected in immunocompromised patients who received neuraminidase inhibitors, and all but 1 had the H275Y neuraminidase mutation (*2*). This mutation had already been observed before the pandemic (H1N1) 2009 outbreak, for example, it was detected worldwide in healthy patients who had not received antiviral drugs and were infected with seasonal (H1N1) virus during the 2008–09 influenza season (*8*). Efforts are under way to characterize and detect the H275Y mutation, but data on clinical impact and viral fitness (i.e., replicative capacity in vitro and in vivo that can further be correlated with transmissibility and virulence) associated with this mutation are still needed. We describe in detail the clinical features, treatment, outcomes, and virologic data associated with infection caused by pandemic (H1N1) 2009 virus with H275Y mutation in immunocompromised patients.

## Materials and Methods

Hematology–oncology patients who were infected with pandemic (H1N1) 2009 virus and received care at adult and pediatric Seattle Cancer Care Alliance (Seattle, WA, USA) units or clinics during May 1, 2009–April 30, 2010, were identified by using infection control data and laboratory databases. All samples with pandemic (H1N1) 2009 virus detected by in-house real-time reverse transcription–PCR targeting the matrix, and hemagglutinin genes were retrospectively tested by our allele-specific real-time PCR (ASPCR) for H275Y genotype (*9**,**10*). ASPCR uses 2 allele-specific forward primers (wild-type and mutant) and a common reverse primer and probe. Wild-type and mutant genotypes were defined by the difference in PCR cycle threshold values (∆cycle threshold_mutant –wild type_) between the mutant primer and the wild-type primer amplification curves for the same sample. The ASPCR directed toward the H275Y mutation only was designed and validated in our laboratory, with good correlation demonstrated by pyrosequencing (*9*). ASPCR also can provide an accurate quantitative result of mutant percentage in a mixed population, as described (*11*). Samples were not tested for adamantanes resistance because pandemic (H1N1) 2009 virus was considered to be uniformly resistant to adamantanes.

Specimens collected from inpatients or outpatients were either nasal wash (NW) samples or bronchoalveolar lavage (BAL) samples. NW samples were tested by using multiplex real-time reverse transcription–PCR for respiratory syncytial virus, parainfluenza virus 1–4, human metapneumovirus, adenovirus, bocavirus, coronavirus (OC43, 229E, HKU1, and NL63), and rhinovirus at the same time as influenza A subtyping and influenza B. BAL samples were tested for the same respiratory viruses and for bacterial, mycobacterial, viral, and fungal cultures, fungal PCR, galactomanan, cytomegalovirus by shell vial, respiratory syncytial virus by shell vial, respiratory virus direct immunofluorescence assay, and *Pneumocystis* spp. direct immunofluorescence assay. NW samples were collected by instilling 5 mL of normal saline in each nare and having the patient blow his or her nose directly into a sterile cup. For younger children, suction was used to collect nasal secretions. BAL samples were obtained according to a standardized protocol. Samples were refrigerated within 4 h after collection and transported to the molecular virology laboratory. All available samples were kept frozen at –80°C for up to 8 months after initial clinical testing. Total nucleic acid was extracted as described (*12*). Institutional review board approval was obtained from our institutional committee. A chart review of all patients with mutant H275Y virus was retrospectively performed by using standardized case record forms.

## Results

### ASPCR Results

We identified 49 adult and pediatric hematology–oncology or hemopoietic cell transplant (HCT) patients who were infected by pandemic (H1N1) 2009 virus during May 1, 2009–April 30, 2010. Of these patients, 16 had no specimen available for genotyping of position H275 by ASPCR because the initial diagnostic test was performed in another laboratory (12 patients), no residual sample was available (3 patients), or the viral load was too low to genotype (1 patient). For 33 patients, at least their first sample, obtained before treatment, was available for genotyping. One of the 33 first samples collected had the H275Y mutation. For 17 patients, repeat samples were obtained for clinical reasons, but only 12 patients had sufficient viral load for genotyping. The H275Y mutation developed in 7 (58%) patients. The H275Y mutation was identified in 8 patients; 3 of these patients (patients 1, 2, and 6) have been described (*5**,**13*).

### Clinical Characteristics

Five of the 8 patients with H275Y mutation had undergone allogenic HCT or were receiving conditioning for future allogenic HCT (Table 1). Two patients had malignancies (acute lymphoblastic leukemia and osteosarcoma), and 1 had aplastic anemia. Four were children and 4 were adults. Three patients had severe lymphopenia, with lymphocyte counts persistently <200 × 10^3^ cells/L. Clinical characteristics are presented in Table 2; viral kinetics and treatment are presented in the Figure for each patient. No patients had contact with another infected patient in the hospital or the clinic, and no evidence was found of nosocomial transmission of resistant strains.

**Table 1:** Demographic characteristics and underlying conditions in 8 patients with H275Y mutation of pandemic (H1N1) 2009 virus, Seattle Cancer Care Alliance, Seattle, Washington, USA, May 1, 2009–April 30, 2010*

Patient no.	Age, y/sex	BMI	Underlying disease	State of disease	HCT	Recent immunosuppressive therapy	Concurrent illness	Lymphocytes, × 10^3^ cells/L
1†	51/M	20.5	AML	Remission	2 y post–allo-HCT	MMF, tacrolimus	GVHD, renal failure	110
2†	47/F	22.9	AML	Relapse	3 y post–allo-HCT	Chemotherapy 1 mo before influenza dx and 1 d after-dx	None	540
3	50/M	23.3	CML	Remission	9 mo post–allo-HCT	TBI, cyclophosphamide, tacrolimus	GVHD	1,470
4	7/F	18.9	ALL	Relapse	NA	Chemotherapy 2 d after influenza diagnosis; prednisone 1.5 mg/kg/d	None	1,440
5	34/F	38.8	Hogkin lymphoma	Refractory	2 d pre–allo-HCT	TBI, fludarabine, cyclophosphamide	Renal failure	0
6†	17/M	19.5	ALL (T cell)	Remission	3 wk post–allo-HCT	TBI, fludarabine, cyclophosphamide, MMF, CSA, prednisone 0.5 mg/kg/d	CMV, GVHD	154
7	17/M	22.4	Osteosarcoma	Relapse	NA	Chemotherapy 1 d before influenza diagnosis	Lung metastasis, thoracotomy, restrictive syndrome	366
8	8/F	14.5	Aplastic anemia	Treatment	NA	ATG, CSA maintenance	None	1,145

*BMI, body mass index; HCT, hemopoietic cell transplant; AML, acute myeloblastic leukemia; allo, allogenic; MMF, mycophenolate mofetil; GVHD, graft-versus-host disease; NA, not applicable; CML, chronic myeloblastic leukemia; TBI, total body irradiation; ALL, acute lymphoblastic leukemia; CSA, cyclosporine; CMV, cytomegalovirus; ATG, antithymocyte globuline. †Patients previously reported in references (*5*,*13*).

**Table 2:** Clinical characteristics and outcomes of 8 patients with H275Y mutation of pandemic (H1N1) 2009 virus, Seattle Cancer Care Alliance, Seattle, Washington, USA, May 1, 2009–April 30, 2010*

Patient no.	Symptoms of URTI	Signs of LRTI	Radiology results	Antiviral drug therapy before resistance	Co-pathogens	Outcome
1	24 h before diagnosis: congestion, headache	Hypoxemia; positive BAL result on d 5	Bilateral ground glass opacity	Oseltamivir 150 mg 2×/d followed by peramivir	None	Death related to influenza
2	48 h before diagnosis: congestion, wet cough, sore throat, fever (24 h)	Hypoxemia; positive BAL result on d 25	Bilateral ground glass opacity	Oseltamivir 150 mg 2×/d + rimantadine 100 mg 2×/d	*Pneumocystis* spp. (DFA + in BAL)	Alive
3	5 d before diagnosis: fever (24 h), wet cough	Hypoxemia; positive BAL result on d 2	Multiple nodules with halo sign	Oseltamivir 150 mg 2×/d	Aspergillus (PCR and GM result positive in BAL sample); *Staphylococcus aureus*; PIV3	Alive
4	<24 h before diagnosis: fever (5 d), cough	Hypoxemia	Bilateral infiltrates	Oseltamivir 2 mg/kg 2×/d	None	Alive
5	<24 h before diagnosis: cough, rhinorrhea, congestion	No	Bronchial thickening	Oseltamivir 150 mg 2×/d	None	Alive
6	<24 h before diagnosis: rhinorrhea, fever (24 h), cough	No	None	Oseltamivir 150 mg 2×/d	None	Alive
7	24 h before diagnosis: sore throat, fever (24 h), cough	No	CXR stable	Oseltamivir 75 mg 2×/d + rimantadine 100 mg 2×/d	Rhinovirus (PCR result positive in NW sample)	Alive
8	24 h before diagnosis: sore throat, fever (24 h), chills	No	CXR normal	Prophylaxis: oseltamivir 45 mg 2×/d for 10 d, ended 3 d before influenza diagnosis	None	Alive

*URTI, upper respiratory tract infection; LRTI, lower respiratory tract infection; BAL, bronchoalveolar lavage; DFA, direct immunofluorescence assay; GM, galactomanane; PIV3, parainfluenza virus 3; NW, nasal wash; CXR, chest radiograph.

**Figure:** Viral kinetics in nasal washes and treatment data of 7 patients (patient nos. 1–7 shown top to bottom) with H275Y mutation of pandemic (H1N1) 2009 virus, Seattle Cancer Care Alliance, Seattle, Washington, USA, May 1, 2009–April 30, 2010. Black lines, pandemic (H1N1) 2009 viral load; red line, % H275Y mutant. OTV, oseltamivir; RBV, ribavirin; RTM, rimantadine; ZNV, zanamivir; PMV, peramivir; BAL, bronchoalveolar lavage.

#### Severe Pneumonia

Severe pneumonia followed by acute respiratory distress syndrome developed in 1 of the 8 patients (patient 1). This patient was initially treated with oseltamivir for 4 days; then treatment was changed to intravenous peramivir because of the patient’s inability to tolerate oral therapy. Just before initiation of peramivir, a BAL sample showed the absence of H275Y mutation in the viral population that were present in the lung but no concomitant NW sample was available for testing. After 7 days of intravenous peramivir, an NW sample showed H275Y mutation in 100% of the viral population. This patient subsequently received triple-combination antiviral drug therapy (i.e., oseltamivir, rimantadine, and oral ribavirin) while awaiting intravenous zanamivir that was administered during days 18–25. The patient died of severe pneumonia and multiorgan failure after several days of mechanical ventilation. Autopsy showed necrotizing pancreatitis with bilateral pulmonary consolidation, pulmonary hemorrhage, diffuse alveolar damage, and patchy fibrosis. Pandemic (H1N1) 2009 virus was proven by PCR in the NW sampled at autopsy but not in the lung tissue or pancreas. Influenza viruses detected in the NW samples were completely wild-type at autopsy.

#### Mild Lower Respiratory Tract Infection with Prolonged Shedding

In 3 patients (patients 2–4), lower respiratory tract infection quickly developed, but the patients recovered without complications. Two of these patients initially were treated with oseltamivir alone, and 1 was treated with oseltamivir and rimantadine. Viral H275Y mutation was detected at days 23, 8, and 17 for patients 2, 3, and 4, respectively. All 3 patients had prolonged mild upper respiratory tract symptoms, consisting mainly of rhinorrhea and dry cough, accompanying a variable duration of viral shedding. Influenza virus was detected in the NW sample for 93, 8, and 47 days in patients 2, 3, and 4, respectively. Patient 2 had shedding of fully resistant viral population documented for at least 26 days after antiviral drug therapy was stopped. Patient 3 recovered after 17 days of oseltamivir therapy, even though a small percentage of H275Y mutations (10%) were present in his NW sample at day 8. In patient 4, the percentage of H275Y mutants declined slightly after treatment was stopped. Two of these 3 patients had substantial concurrent pathogens (*Pneumocystis jiroveci* and *Aspergillus fumigatus*) in BAL samples at the same time as pandemic (H1N1) 2009 virus infection, making the diagnosis of pandemic (H1N1) 2009–related pneumonia less certain.

#### Upper Respiratory Tract Infection with Prolonged Shedding

Three patients (patients 5–7) had symptoms of only upper respiratory tract disease. None had hypoxemia or infiltrate on chest radiograph, but all 3 had a cough and 2 had fever for 24 hours. They all had profound lymphopenia (0–366 × 10^3^ cells/L) and 1 had graft-versus-host disease. Two patients were initially treated with oseltamivir alone and 1 with oseltamivir and rimantadine. One hundred percent mutant virus developed in patients 5–7; these patients shed influenza virus for 65, 75, and 12 days, respectively. Patient 5 maintained a 100% mutant viral population while receiving peramivir and continued to maintain this percentage while on prolonged oseltamivir therapy. Patient 6 remained infected with a fully resistant viral population many days after oseltamivir was stopped. Patient 7 improved rapidly, but because of underlying lung disease, PCR was repeated on day 12 and showed continual viral shedding with a low viral load. No other viral testing was performed, and this patient recovered completely with a second 5-day course of oseltamivir.

#### Prophylaxis

One young patient (patient 8) had 85% H275Y mutant viral population detected in the first NW sample obtained on day 2 of illness. This patient had been in contact with 2 family members who had documented influenza infection but did not receive antiviral drug therapy. The patient then received oseltamivir prophylaxis (45 mg 2×/d) for 10 days; 3 days after prophylaxis was completed symptoms developed, including fever, chills and sore throat. The patient was treated with 10 days of oseltamivir and had a mild course of disease with rapid clinical resolution.

### Initial Therapy

In all patients, except patient 8, virus was fully wild-type when the initial specimen was collected. All patients had received antiviral drug therapy before the H275Y mutation was detected. One patient had received oseltamivir prophylaxis, 4 had received only oseltamivir treatment, 2 had received combination oseltamivir/rimantadine therapy, and 1 had received oseltamivir followed by intravenous peramivir.

### Virologic Data

In 3 of the 8 patients (patients 4–6), viral loads increased after H275Y viruses were detected. In 2 patients (patients 2 and 4), viral loads declined after start of either intravenous or inhaled zanamivir. In 1 patient, viral load declined during 17 days of oseltamivir therapy, even though 10% of the viral population was documented to be H275Y mutants. Five of the 8 patients were found at some time to have mixed, wild-type and mutant, populations. We did not identify any discrepancy between BAL sample and NW sample genotyping results, but both types of samples were collected in close proximity (within 2–4 days) in only 3 patients. Wild-type virus was detected in 2 patients in paired NW and BAL samples; mutant virus was detected in paired NW and BAL samples in 1 patient. The 3 patients with the highest initial viral load (>6 log_10_ copies/reaction) had a substantial percentage of H275Y mutants at days 8, 9, and 11 (range 35%–100%). Patients with lower initial viral loads were not tested a second time until 12–23 days later. These long intervals do not enable us to compare the timing of resistance emergence according to initial viral load.

## Discussion

During the pandemic (H1N1) 2009 outbreak, neuraminidase-resistant viruses emerged rapidly in immunocompromised patients. Our retrospective analysis suggests a high rate of oseltamivir resistance conferred by the H275Y mutation in treated immunocompromised patients. The rate of mutation development ranged from 8 (16%) of 49 patients, with all infected patients as denominator, to 7 (58%) of 12 on the basis of samples obtained after antiviral drug therapy began. Two other studies have reported similar rates. One study from Scotland reported a 50% rate (5/10) of H275Y mutation in immunocompromised patients with samples available after oseltamivir therapy began (*14*). The other study, in Australia, reported a 13.3% rate (4/30) of H275Y mutation in treated immunocompromised patients (not all of them tested) or 57% rate (4/7) in only treated patients for whom samples were available after treatment began (*15*).

The 8 patients in our study who had H275Y mutant virus demonstrated a wide range of clinical disease, from benign upper respiratory tract symptoms to severe and fatal respiratory insufficiency. Whether the H275Y mutation is associated with higher rates of death or severe disease is unclear. The small number of immunocompromised patients infected with resistant pandemic (H1N1) 2009 virus did not enable us to compare them with patients infected with sensitive pandemic (H1N1) 2009 virus. However, our case series highlights the rapid emergence of resistant viruses in the context of mild to severe influenza disease. In 7 of the 8 cases in our study, resistance was not associated with long-term consequences and was not even suspected in 4 of the 7 cases. This observation could suggest that resistant viruses are not more virulent than wild-type viruses.

Influenza resistance kinetics as provided in this study by the percentage of H275Y mutants enable a better determination of the timing of emergence of resistant virus and the possible subsequent clearance of this resistant viral population. Our results suggest that elevated viral loads seen early in disease might be associated with more rapid emergence of resistance, but more complete and regular testing after the initial sample is necessary to confirm that hypothesis.

In this case series, some patients conserved the resistant viral population after antiviral pressure removal, whereas another patient (patient 1) cleared his resistant viral population to recover a fully wild-type virus. Similarly, the H275Y mutation described in another case report disappeared after antiviral drug therapy was completed, while others showed conserved resistant viral population (*14**,**16*). Those 2 different evolutions of mutant viral population suggest the possibility of quasispecies with different fitness. The H275Y neuraminidase mutation had affected in vitro viral fitness when incorporated in seasonal (H1N1) virus, but data looking at 2008–09 seasonal (H1N1) virus showed that the circulating H275Y mutant strain had recovered its fitness, possibly explaining its sustainable transmission (*17*). Recently, permissive secondary mutations in the neuraminidase gene leading to fitness recovery have been proposed in seasonal (H1N1) viruses (*18*). Substitutions V234M and R222Q buffer deficiencies in neuraminidase folding or stability caused by H275Y and simultaneously allow the virus to keep its neuraminidase/hemagglutinin balance, which is implicated in viral fitness (*19*). Further molecular and in vitro analysis of viral strains from patients who cleared and did not clear the mutant viral population might provide more input about permissive secondary mutations in pandemic (H1N1) 2009 virus.

We were not able to provide information about whether an H275Y mutant viral population can develop in the upper respiratory tract and not in the lower respiratory tract or the converse scenario. The limited data we described on paired NW and BAL samples suggest that the upper and lower respiratory tracts are likely to be infected with the same viral H275Y genotype. Others have shown that quasispecies harboring the D222G hemagglutinin mutation that confers improved pneumocyte receptors binding could be found specifically or in larger proportions in endotracheal aspirates than in paired nasopharyngeal aspirates (*20*). Analysis of D222G mutant virus has not yet been reported in immunocompromised patients, a condition in which mutations develop more readily.

We have demonstrated that the H275Y mutation can develop in immunocompromised patients under different antiviral drug treatment regimens. Oseltamivir is the antiviral agent most associated with the emergence of H275Y. Peramivir selects for the H275Y mutation in seasonal (H1N1) by successive passages in vitro, but data have not clearly confirmed this observation in vivo (*21*). One patient in our study had received oseltamivir and peramivir before resistance detection. We believe that if peramivir was not the drug that selected the initial mutation, it did not prevent establishment of dominant H275Y virus population (*13*). Because peramivir is rarely used as frontline therapy, data to support this hypothesis are likely to be difficult to obtain. Combination therapy with oseltamivir and rimantadine did not prevent development of H275Y mutation in 2 of the patients in our study. The issue of antiviral drug resistance in immunocompromised patients makes determination of optimal initial therapy necessary. Inhaled zanamivir is often contraindicated because the patients may have respiratory failure or underlying lung disease, and novel agents (e.g., DAS181) are not yet available. Triple-combination therapy (i.e., oseltamivir, rimantadine, and oral ribavarin) potentially could reduce emergence of resistance, but more data are needed to support this hypothesis (*22**,**23*).

This case series has some limitations. It does not provide a totally accurate incidence of resistance in immunocompromised patients. Some cases of pandemic (H1N1) 2009 might have been missed, particularly if symptoms were mild or if the patient was not identified by infection control surveillance. Also, repeat testing was performed in only 17 patients, in whom 5 viral loads were too low to genotype. Systematic testing of infected patients at 5 or 6 days after initial pandemic (H1N1) 2009 diagnosis might have caught even more minor mutant viral populations. Our ASPCR was aimed only at detection of the H275Y mutation. Presence of other rare mutations as the I223R could have influenced our results (*24**,**25*). However, our data highlight the need for surveillance and clinical testing for resistant mutations in immunocompromised patients by using sensitive and rapid molecular diagnostic tests.

## References

[1] Lackenby A, Thompson CI, Democratis J. The potential impact of neuraminidase inhibitor resistant influenza. Curr Opin Infect Dis. 2008;21:626–38. 10.1097/QCO.0b013e328319979718978531

[2] World Health Organization. Weekly update on oseltamivir resistance to pandemic influenza A (H1N1) 2009 viruses [cited 2010 Jul 14]. http://www.who.int/csr/disease/swineflu/laboratory16_07_2010/en/index.html

[3] Memoli MJ, Hrabal RJ, Hassantoufighi A, Eichelberger MC, Taubenberger JK. Rapid selection of oseltamivir- and peramivir-resistant pandemic H1N1 virus during therapy in 2 immunocompromised hosts. Clin Infect Dis. 2010;50:1252–5. 10.1086/65160520345239PMC2946636

[4] Gulland A. First cases of spread of oseltamivir resistant swine flu between patients are reported in Wales. BMJ. 2009;339:b4975. 10.1136/bmj.b497519933735

[5] Centers for Disease Control and Prevention. Oseltamivir-resistant novel influenza A (H1N1) virus infection in two immunosuppressed patients—Seattle, Washington, 2009. MMWR Morb Mortal Wkly Rep. 2009;58:893–6.19696719

[6] Balicer RD, Huerta M, Davidovitch N, Grotto I. Cost-benefit of stockpiling drugs for influenza pandemic. Emerg Infect Dis. 2005;11:1280–2.1610231910.3201/eid1108.041156PMC3320484

[7] Lee VJ, Yap J, Cook AR, Chen MI, Tay JK, Tan BH, Oseltamivir ring prophylaxis for containment of 2009 H1N1 influenza outbreaks. N Engl J Med. 2010;362:2166–74. 10.1056/NEJMoa090848220558367

[8] Moscona A. Global transmission of oseltamivir-resistant influenza. N Engl J Med. 2009;360:953–6. 10.1056/NEJMp090064819258250

[9] Renaud C, Kuypers J, Corey L. Diagnostic accuracy of an allele-specific reverse transcriptase–PCR assay targeting the H275Y oseltamivir resistant mutation in 2009 pandemic influenza A/H1N1 virus. J Clin Virol. 2010;49:21–5. 10.1016/j.jcv.2010.06.01920674476

[10] Kuypers J, Wright N, Ferrenberg J, Huang ML, Cent A, Corey L, Comparison of real-time PCR assays with fluorescent-antibody assays for diagnosis of respiratory virus infections in children. J Clin Microbiol. 2006;44:2382–8. 10.1128/JCM.00216-0616825353PMC1489473

[11] Liu CM, Driebe EM, Schupp J, Kelley E, Nguyen JT, McSharry JJ, Rapid quantification of single-nucleotide mutations in mixed influenza A viral populations using allele-specific mixture analysis. J Virol Methods. 2010;163:109–15. 10.1016/j.jviromet.2009.09.00719761797

[12] Kuypers J, Wright N, Morrow R. Evaluation of quantitative and type-specific real-time RT-PCR assays for detection of respiratory syncytial virus in respiratory specimens from children. J Clin Virol. 2004;31:123–9. 10.1016/j.jcv.2004.03.01815364268PMC7128826

[13] Renaud C, Pergam SA, Polyak C, Jain R, Kuypers J, Englund JA, Early emergence of an H275Y mutation in a hematopoietic cell transplant recipient treated with intravenous peramivir. Transpl Infect Dis. 2010;12:513–7. 10.1111/j.1399-3062.2010.00582.x21062390PMC3024056

[14] Harvala H, Gunson R, Simmonds P, Hardie A, Bennett S, Scott F, The emergence of oseltamivir-resistant pandemic influenza A(H1N1) 2009 virus amongst hospitalised immunocompromised patients in Scotland, November–December, 2009. Euro Surveill. 2010;15: pii: 19536.20394718

[15] Tramontana AR, George B, Hurt AC, Doyle JS, Langan K, Reid AB, Oseltamivir resistance in adult oncology and hematology patients infected with pandemic (H1N1) 2009 virus, Australia. Emerg Infect Dis. 2010;16:1068–75. 10.3201/eid1607.09169120587176PMC3321901

[16] Campanini G, Piralla A, Rovida F, Puzelli S, Facchini M, Locatelli F, First case in Italy of acquired resistance to oseltamivir in an immunocompromised patient with influenza A/H1N1v infection. J Clin Virol. 2010;48:220–2. 10.1016/j.jcv.2010.03.02720447860

[17] Baz M, Abed Y, Simon P, Hamelin ME, Boivin G. Effect of the neuraminidase mutation H274Y conferring resistance to oseltamivir on the replicative capacity and virulence of old and recent human influenza A(H1N1) viruses. J Infect Dis. 2010;201:740–5. 10.1086/65046420100088

[18] Bloom JD, Gong LI, Baltimore D. Permissive secondary mutations enable the evolution of influenza oseltamivir resistance. Science. 2010;328:1272–5. 10.1126/science.118781620522774PMC2913718

[19] Wagner R, Matrosovich M, Klenk HD. Functional balance between haemagglutinin and neuraminidase in influenza virus infections. Rev Med Virol. 2002;12:159–66. 10.1002/rmv.35211987141

[20] Chen H, Wen X, To KK, Wang P, Tse H, Chan JF, Quasispecies of the D225G substitution in the hemagglutinin of pandemic influenza A(H1N1) 2009 virus from patients with severe disease in Hong Kong, China. J Infect Dis. 2010;201:1517–21. 10.1086/65266120367331

[21] Baz M, Abed Y, Boivin G. Characterization of drug-resistant recombinant influenza A/H1N1 viruses selected in vitro with peramivir and zanamivir. Antiviral Res. 2007;74:159–62. 10.1016/j.antiviral.2006.10.01217137644

[22] Nguyen JT, Hoopes JD, Smee DF, Prichard MN, Driebe EM, Engelthaler DM, Triple combination of oseltamivir, amantadine, and ribavirin displays synergistic activity against multiple influenza virus strains in vitro. Antimicrob Agents Chemother. 2009;53:4115–26. 10.1128/AAC.00476-0919620324PMC2764153

[23] Nguyen JT, Hoopes JD, Le MH, Smee DF, Patick AK, Faix DJ, Triple combination of amantadine, ribavirin, and oseltamivir is highly active and synergistic against drug resistant influenza virus strains in vitro. PLoS ONE. 2010;5:e9332. 10.1371/journal.pone.000933220179772PMC2825274

[24] Nguyen HT, Fry AM, Loveless PA, Klimov AI, Gubareva LV. Recovery of a multidrug-resistant strain of pandemic influenza A 2009 (H1N1) virus carrying a dual H275Y/I223R mutation from a child after prolonged treatment with oseltamivir. Clin Infect Dis. 2010;51:983–4. 10.1086/65643920858074

[25] van der Vries E, Stelma FF, Boucher CA. Emergence of a multidrug-resistant pandemic influenza A (H1N1) virus. N Engl J Med. 2010;363:1381–2. 10.1056/NEJMc100374920879894
